# Polyhydramnios among women in a cluster-randomized trial of ultrasound during prenatal care within five low and low-middle income countries: a secondary analysis of the first look study

**DOI:** 10.1186/s12884-019-2412-6

**Published:** 2019-07-22

**Authors:** Melissa Bauserman, Robert Nathan, Adrien Lokangaka, Elizabeth M. McClure, Janet Moore, Daniel Ishoso, Antoinette Tshefu, Lester Figueroa, Ana Garces, Margo S. Harrison, Dennis Wallace, Sarah Saleem, Waseem Mirza, Nancy Krebs, Michael Hambidge, Waldemar Carlo, Elwyn Chomba, Menachem Miodovnik, Marion Koso-Thomas, Edward A. Liechty, Fabian Esamai, Jonathan Swanson, David Swanson, Robert L. Goldenberg, Carl Bose

**Affiliations:** 1 Department of Pediatrics, University of North Carolina School of Medicine, 101 Manning Drive, CB 7596, Chapel Hill, NC 27599-7596 USA; 2 Department of Radiology, Harborview Medical Center, University of Washington Medical Center, Seattle, WA USA; 3 Kinshasa School of Public Health, Kinshasa, DRC Republic of the Congo; 4 RTI International, Durham, NC USA; 5 Fundación para la Alimentación y Nutrición de Centro América y Panamá (FANCAP), Guatemala City, Guatemala; 6 Department of Obstetrics and Gynecology, University of Colorado, Denver, CO USA; 7 Department of Community Health Sciences, Aga Khan University, Karachi, Pakistan; 8 Department of Pediatric Radiology, Aga Khan University, Karachi, Pakistan; 9 Department of Pediatrics, University of Colorado, Denver, CO USA; 10 Department of Pediatrics, University of Alabama at Birmingham, Birmingham, AL USA; 11 Department of Pediatrics, University of Zambia, Lusaka, Zambia; 12 Perinatology and Pregnancy Branch, Eunice Kennedy Shriver National Institute of Child Health and Human Development, Rockville, MD USA; 13 Department of Pediatrics, Indiana University, Indianapolis, IN USA; 14 School of Medicine, Moi University, Eldoret, Kenya; 15 Department of Radiology, Seattle Children’s Hospital, University of Washington Medical Center, Seattle, WA USA; 16 Department of Radiology, University of Washington Medical Center, Seattle, WA USA; 17 Department of Obstetrics/Gynecology, Columbia University, New York City, NY USA

**Keywords:** Polyhydramnios, Low-income country, Global health

## Abstract

**Background:**

In many low and low-middle income countries, the incidence of polyhydramnios is unknown, in part because ultrasound technology is not routinely used. Our objective was to report the incidence of polyhydramnios in five low and low-middle income countries, to determine maternal characteristics associated with polyhydramnios, and report pregnancy and neonatal outcomes.

**Methods:**

We performed a secondary analysis of the First Look Study, a multi-national, cluster-randomized trial of ultrasound during prenatal care. We evaluated all women enrolled from Guatemala, Pakistan, Zambia, Kenya and the Democratic Republic of Congo (DRC) who received an examination by prenatal ultrasound. We used pairwise site comparisons with Tukey-Kramer adjustment and multivariable logistic models with general estimating equations to control for cluster-level effects. The diagnosis of polyhydramnios was confrimed by an U.S. based radiologist in a majority of cases (62%).

**Results:**

We identified 305/18,640 (1.6%) cases of polyhydramnios. 229 (75%) cases were from the DRC, with an incidence of 10%. A higher percentage of women with polyhydramnios experienced obstructed labor (7% vs 4%) and fetal malposition (4% vs 2%). Neonatal death was more common when polyhydramnios was present (OR 2.43; CI 1.15, 5.13).

**Conclusions:**

Polyhydramnios occured in these low and low-middle income countries at a rate similar to high-income contries except in the DRC where the incidence was 10%. Polyhydramnios was associated with obstructed labor, fetal malposition, and neonatal death.

**Trial registration:**

NCT01990625, November 21, 2013.

## Background

Polyhydramnios, or an excessive accumulation of amniotic fluid, affects less than 2% of pregnancies in high-income countries [1, 2]. Polyhydramnios can be associated with adverse outcomes such as perinatal mortality, prematurity, shoulder dystocia, and respiratory distress syndrome, and is therefore an important health concern [3–5]. Polyhydramnios can result from maternal conditions such as poorly controlled diabetes, infections and medication exposure [4]. Fetal conditions that affect the fetus’s ability to swallow or obstructive gastrointestinal pathologies can also result in polyhydramnios [4].

In many low and low-middle income countries (LMICs), the incidence of polyhydramnios is unknown. The use of ultrasound during prenatal care is limited in many LMICs due to the high cost of purchasing and maintaining ultrasound equipment, and lack of trained providers to perform and interpret these studies. As a result, certain pregnancy-related conditions that are usually detected by ultrasound go undiagnosed, including polyhydramnios, oligohydramnios and fetal growth abnormalities [6]. These conditions can lead to adverse perinatal events, and therefore should be recognized in order to improve maternal and neonatal outcomes. Maternal and neonatal outcomes associated with polyhydramnios may be worse in LMICs due to limitations in emergent obstetric care and intensive care for infants born prematurely. Understanding the epidemiology of polyhydramnios in LMICs might lead to strategies for reducing perinatal and peripartum morbidity and mortality.

Pregnant women in LMICs may have different underlying risk factors for polyhydramnios when compared to women in high-income countries. Women in LMICs have different nutritional status, infection exposures, prevalence of diabetes and incidence of fetal anomalies than women in high-income countries [2, 7–10]. These differences in exposure might alter the prevalence of polyhydramnios in these regions. These maternal characteristics might also contribute to worse perinatal outcomes.

In this paper, we report the incidence of polyhydramnios among women in five LMICs. We also report maternal characteristics associated with polyhydramnios and the pregnancy and neonatal outcomes among those affected.

## Methods

We evaluated all women enrolled in the First Look Study who received one or more prenatal ultrasounds. The details of the First Look Study have been described elsewhere, but briefly, this was a multi-country study conducted from July 2014 to May 2016 to determine if the use of ultrasound technology during prenatal care in LMICs improved maternal mortality, maternal near-miss mortality, stillbirth and neonatal mortality [6, 11]. The First Look Study was a cluster randomized trial in rural areas within five LMICs: Guatemala, Democratic Republic of Congo (DRC), Zambia, Pakistan and Kenya. Women living in the intervention clusters were asked to receive two ultrasound examinations at antenatal care visits, the first when pregnancy was identified, ideally between 16 and 22 weeks, and the second at 32–36 weeks of gestation. Many of the first ultrasounds were performed after the ideal gestational window, but most of the second ultrasounds were preformed within the ideal window. The first ultrasound was used to estimate gestational age, fetal number and position, amniotic fluid abnormalities, cervical length and major congenital anomalies. The second ultrasound evaluated placental location, fetal growth, fetal position, amniotic fluid abnormalities, and major congenital anomalies.

Medical officers, nurses, midwives, and radiographers with no prior ultrasound experience were trained in the use of obstetric ultrasound during a two-week training period under the supervision of a radiologist (RN) based at the University of Washington, Seattle, WA, USA, using the curriculum based from the Basic Obstetric Ultrasound Training Instructor Guide and Basic Obstetric Ultrasound Participant Manual, developed at the University of Washington [12]. All trainees were evaluated using pre- and post written and practical scanning tests. Central trainers evaluated the skills of all trainess and certified those who successfully completed the course. All sites successfully completed a three-month pilot period to evaluate the sonographers’ ability and the integration of the study procedures into the context of the local health care system before inclusion in the study. Throughout the trial, quality asurance (QA) procedures were in place to maintain appropriate accuracy of ultrasound diagnosis. These QA procedures included uploading of ultrasound images to a website for periodic review of field ultrasound, and central trainers working on site with study staff to periodically evaluate the quality of images produced by community ultrasonographers. Refresher training sessions lasting 2–3 days were held approximately six months after the initial two week ultrasound training.

We defined polyhydramnios using the following criteria: a maximum vertical pocket (MVP) of 8 cm or greater and amniotic fluid index (AFI) of 24 cm or greater. MVP was used for twins or for fetuses less than 24 weeks gestational age (GA), and AFI for GA of 24 weeks or greater. We considered a woman to have polyhydramnios if polyhydramnios was identified on any of the ultrasounds during her pregnancy. A single reviewer at the University of Washington (RN) reviewed a sample of 62% of cases polyhydramnios to ensure accuracy of diagnosis.

In this secondary analysis, we included all women from the First Look study who completed the study and had delivery outcomes recorded. We excluded maternal or fetal deaths that occurred prior to 20 weeks. Because the prevalence of polyhydramnios was higher in the DRC than the other sites, we first determined if the incidence of polyhydramnios in the DRC was statistically different from each of the other sites, and then verified that the incidence at the other sites was not statistically different from each other. We obtained mean differences from a logistic model evaluating women with polyhydramnios, using general estimating equations (GEE) to control for cluster level effects within countries with site included as an explanatory variable. We obtained means from the model for all pairwise site comparisons and *p*-values with a Tukey-Kramer adjustment for multiple comparisons. Because the DRC was significantly different from all other sites with adjusted p-values < 0.001 for all comparisons, but we found minimal difference among other sites, we adjusted all subsequent models for the DRC compared with all other sites.

To determine the maternal characteristics associated with polyhydramnios a priori, we built a regression model. To evaluate the maternal characteristics, we built individual logistic models for each characteristic and the finding of polyhydramnios with GEE to control for cluster level effects, and DRC/other site and the characteristic included as explanatory variables. We then built a multivariable logistic model for polyhydramnios and all maternal characteristics that had an association with polyhydramnios (*p* < 0.10) as explanatory models, using GEE extensions. We excluded maternal weight from this model due to 16% missing values from Kenya and measurement variability across multiple different time points in gestation across sites. The final model included the following maternal characteristics: maternal age, parity, and previous live birth. Due to small number of cases and to avoid multiple testing, we did not analyze maternal characterisitics in each of the five LMICs separately.

We then determined the risk of selected outcomes adjusting for polyhydramnios, DRC/other site and the maternal characteristics evaluated in the previous multivariable model. For binomial outcomes, we created a multivariable logistic model with GEE extensions to control for cluster level effects, DRC/other site, maternal characteristics and polyhydramnios. We report the odds ratio and 95% confidence intervals. For continuous outcomes, we created a regression model with GEE extensions to control for cluster level effects, DRC/other site, maternal characteristics and polyhydramnios. For these models, we report the estimates and 95% confidence intervals. We considered *p* < 0.05 significant, beta 0.80. All analyses were performed at the Data Coordinating Center at RTI International (Durham, NC) using SAS, Inc. version 9.4.

At each site, institutional review boards or ethics committees approved the primary study. All women provided written informed consent before the start of the study. A data monitoring committee, appointed by the NICHD oversaw and reviewed the study annually. This study was supported by grants from the Bill & Melinda Gates Foundation, Eunice Kennedy Shriver National Institute of Child Health and Human Development, and by a grant from GE Healthcare.

## Results

We identified 305 (1.6%) cases of polyhydramnios among 18,640 women. The incidence of polyhydramnios ranged from 0.3% in Guatemala to 10% in DRC (Table 1). Of the women identified with polyhydramnios, 229 (75%) were from the DRC. Compared to women without polyhydramnios, women with polyhydramnios were more often older than 35 years (10% vs 7%, *p* < 0.001), had more than 2 previous live births (71% vs 52%, *p* < 0.001), had a previous live birth (98% vs 94%, < 0.001) and lower mean maternal weight (55.8 kg vs 56.5 kg, *p* = 0.005), Table 2. These trends persisted when the DRC data were evaluated separately from the other sites, with the exception of mean maternal weight. In the DRC, the mean maternal weight was higher among the women with polyhydramnios compared to the women without polyhydramnios (54.5 kg vs 53.5 kg). In a multivariable model evaluating maternal characteristics and polyhydramnios, evaluating DRC site vs. other sites, age, parity, previous live birth, and using GEE to control for cluster level effects, the DRC had an odds ratio of 22.6 (14.87, 34.35), (Table 3).

**Table 1:** Incidence of Polyhydramnios by Site

	Overall	DRC	Kenya	Zambia	Guatemala	Pakistan
N	18,640	2,291	3,380	4,408	6,157	2,404
Incidence of Polyhydramnios, n (%)	305 (1.6)	229 (10.0)	15 (0.4)	30 (0.7)	21 (0.3)	10 (0.4)

**Table 2:** Maternal Characteristics by Polyhydramnios

	Overall			DRC		Other Sites	
	With Polyhydramnios	No Polyhydramnios	*P*-value^*^	With Polyhydramnios	No Polyhydramnios	With Polyhydramnios	No Polyhydramnios
Maternal age (years)	305	18,323	0.0009	229	2,062	76	16,261
< 20	36 (12)	3,351 (18)		27 (12)	443 (22)	9 (12)	2,908 (18)
20–35	238 (78)	13,618 (74)		182 (80)	1,482 (72)	56 (74)	12,136 (75)
> 35	31 (10)	1,354 (7)		20 (9)	137 (7)	11 (15)	1,217 (8)
Maternal education, n	305	18,327	0.4834	229	2,062	76	16,265
No formal schooling	93 (31)	3,850 (21)		77 (34)	654 (32)	16 (21)	3,196 (20)
Primary	127 (42)	5,488 (30)		103 (45)	957 (46)	24 (32)	4,531 (28)
Secondary	82 (27)	8,232 (45)		49 (21)	442 (21)	33 (43)	7,790 (48)
University	3 (1)	757 (4)		0 (0)	9 (0)	3 (4)	748 (5)
Parity, n	304	18,055	< 0.0001	229	2,062	75	15,993
0	41 (14)	4,576 (25)		24 (11)	434 (21)	17 (23)	4,142 (26)
1	48 (16)	4,089 (23)		41 (18)	389 (19)	7 (9)	3,700 (23)
2+	215 (71)	9,390 (52)		164 (72)	1,239 (60)	51 (68)	8,151 (51)
Previous live birth, n (%)	258 (98)	12,652 (94)	0.0006	202 (99)	1,582 (97)	56 (97)	11,070 (93)
Maternal height, cm, Mean (std)	157.1 (7.7)	153.5 (8.1)	0.2147	157.7 (7.3)	157.7 (6.5)	154.9 (8.8)	152.9 (8.1)
Maternal weight, kg, Mean (std)	55.8 (9.8)	56.5 (10.2)	0.0045	54.4 (7.5)	53.5 (6.9)	60.0 (14.1)	56.9 (10.4)

^*^P-value from a logistic regression model for polyhydramnios, adjusting for DRC/Other and maternal characteristic with general estimating equations

**Table 3:** Association of maternal characteristics and polyhydramnios

	Odds Ratio (95% CI)*	*P*-value*
Country		< 0.0001
DRC	22.60 (14.87, 34.35)	< 0.0001
Other	Reference	
Maternal age (years)		0.0820
< 20	0.72 (0.52, 0.99)	0.0438
20–35	Reference	
> 35	1.29 (0.94, 1.76)	0.1107
Parity		0.0596
0	1.76 (0.77, 4.02)	0.1803
1	Reference	
2+	1.44 (1.07, 1.93)	0.0176
Previous live birth		
Yes	2.00 (0.91, 4.41)	0.0850
No or Primiparous	Reference	

*From a multivariable logistic regression model for polyhydramnios adjusting for DRC/Other, age, parity and previous live birth with general estimating equations to control for cluster level effects

We observed a higher percentage of women who experienced obstructed labor (7% vs 4%) and fetal malposition (4% vs 2%) among the women with polyhydramnios (Table 4). These trends persisted when DRC was evaluated separately from the other sites. The number of women who delivered by Cesarean section (C-section) was lower among women with polyhydramnios in the overall analysis (6 vs 12%), but when DRC data were evaluated separately from the other sites, a larger portion of women in the DRC with polyhydramnios were delivered by C-section compared to women without polyhydramnios (3 vs 1%). The percentage of women who were delivered by C-section was equivalent in the two groups across other sites (13%).

**Table 4:** Delivery Complications and Fetal/Neonatal Outcomes by Polyhydramnios

	Overall			DRC		Other	
	With Polyhydramnios *N* = 305	No Polyhydramnios *N* = 18,327	Odds Ratio^b^/Estimate^c^ (95% CI)	With Polyhydramnios *N* = 229	No Polyhydramnios *N* = 2062	With Polyhydramnios *N* = 76	No Polyhydramnios *N* = 16,265
Delivery complications							
Obstructed labor, n (%)	20 (7)	773 (4)		12 (5)	43 (2)	8 (11)	730 (5)
Hemorrhage, n (%)	6 (2)	360 (2)		1 (0)	20 (1)	5 (7)	340 (2)
Hypertensive disorder, n (%)	6 (2)	366 (2)		0 (0)	0 (0)	6 (8)	366 (2)
Fetal malposition, n (%)	12 (4)	352 (2)		7 (3)	13 (1)	5 (7)	339 (2)
C-section delivery, n (%)	17 (6)	2,154 (12)		7 (3)	28 (1)	10 (13)	2,126 (13)
Maternal death < 42 days, n (rate/100,000 deliveries)	0 (0)	23 (126)		0 (0)	3 (146)	0 (0)	20 (123)
Maternal sepsis, n (%)	5 (2)	281 (2)		2 (1)	27 (1)	3 (4)	254 (2)
Fetal/Neonatal outcomes^a^							
Male, n (%)	173 (57)	9,314 (51)		137 (60)	1,068 (52)	36 (47)	8,246 (51)
Low birth weight, n (%)	23 (8)	2,288 (13)		15 (7)	226 (11)	8 (11)	2,062 (13)
GA at delivery, Mean (std)	39.0 (2.9)	38.6 (3)		39.1 (3)	38.7 (13)	38.8 (3.4)	38.5 (5)
Multiple gestation, n (%)	1 (0)	205 (1)		0 (0)	42 (2)	1 (1)	163 (1)
Congenital anomaly, n (%)	0 (0)	32 (0)		0 (0)	0 (0)	0 (0)	32 (0)
Stillbirth, n (rate/1,000)	13 (43)	451 (25)	1.29 (0.78, 2.12)	10 (44)	77 (37)	3 (40)	374 (23)
Birth weight, g, Mean (std)	3082 (551)	2979 (498)	79.2 (31.1, 127.2)	3102 (528)	2965 (533)	3023 (615)	2981 (494)
Preterm Birth, n (%)	33 (11)	2,359 (13)	0.89 (0.54, 1.46)	22 (10)	262 (13)	11 (16)	2,097 (13)
Neonatal death < 28 days, n (rate/1,000)	12 (41)	379 (21)	2.43 (1.15, 5.13)	5 (23)	36 (18)	7 (97)	343 (22)

^a^Fetal/Neonatal outcomes are calculated at the maternal level if at least one fetus/neonate has the outcome

^b^Odds Ratio from a multivariable logistic regression model adjusting for at least one polyhydramnios finding, DRC/Other, Age, Parity and Previous LB with general estimating equations to control for cluster level effects

^c^Estimate from a multivariable regression model adjusting for at least one polyhydramnios finding, DRC/Other, Age, Parity and Previous LB with general estimating equations to control for cluster level effects

In a multivariable regression model, adjusting for age, parity, previous live birth and controlling for cluster level effects, the odds ratio for neonatal death in pregnancies that were complicated by polyhydramnios was 2.43 (1.15, 5.13), (Table 4). Neonatal mortality rates were higher in the DRC when polyhydramnios was present (23/1,000 vs 18/1,000). The odds of stillbirth given polyhdramnios was 1.29 (0.78, 2.12). The incidence of preterm birth was not different among pregnancies complicated by polyhydramnios compared to those without polyhydramnios (11% vs 13%), and the odds ratio was not significant when adjusted for the previous variables.

Polyhydramnios was associated with a higher mean birthweight at delivery (3082 g vs 2979 g), although this was not significant in the final model. In the DRC, among pregnancies affected by polyhydramnios, a lower proportion of infants were born with low birth weight (7 vs 11%), (Table 4).

## Discussion

The First Look Study evaluated the effect of introducing ultrasound technology in regions of LMICs where ultrasound had not been a component of prenatal care [11]. The objective of the First Look Study was to determine if introducing prenatal ultrasound to resource-limited areas would reduce maternal mortality, maternal near-miss mortality, stillbirth and neonatal mortality. While the First Look Study did not see a difference in the primary outcome by using prenatal ultrasound, a higher than expected incidence of polyhydramnios was identified. We report the incidence of polyhydramnios in these regions, where data on polyhydramnios is limited. The incidence of polyhydramnios in the DRC was much higher than the incidence reported in high-income countries, and higher than the incidence in the other LMICs included in the First Look Study: Guatemala, Pakistan, Kenya and Zambia. Women with polyhydramnios were older, had more than 2 children, and lower weight. Across all countries, polyhydramnios was associated with adverse pregnancy and birth outcomes such as obstructed labor, fetal malpresentation and neonatal death.

Our study had several key strengths. This is the first study to evaluate prenatal ultrasound in relation to polyhydramnios in these regions of the world. To ensure accurate diagnosis of ultrasound findings, we conducted rigorous training, a pilot trial to ensure accurate results and frequent quality assurance checks throughout the study. Additionally, our ultrasound findings were verified by an independent US-based radiologist. We evaluated all patients in the First Look Study, which enrolled pregnant women within the study communities. This method of data collection permitted population-level conclusions for the communities studied; therefore, our data are representative of these communities. Our study does have some practical limitations. Although this study is representative of the study communities, the results might not be representative of the country as a whole. Furthermore, because our study was a secondary analysis of the First Look Study, we were limited by the data that were collected for the primary study. This limitation prevented us from determining the etiology of polyhydramnios or the effect these underlying pathologies might have had on outcomes.

Our study was not designed to investigate the causal factors for polyhydramnios. Polyhydramnios results from a disruption of the equilibrium between the production and resorption of amniotic fluid. Imbalances could result from increased production of fluid through fetal urination and production of fetal lung fluid or from impaired reabsorption from impaired fetal swallowing and diminished intramembranous and intravascular absorption. Although mild polyhydramnios is often idiopathic, reported causes of moderate and severe polyhydramnios include: fetal malformations and genetic anomalies (8–45%), maternal diabetes mellitus (5–26%), multiple pregnancies (8–10%), fetal anemia (1–11%), and other causes, including viral infections, Bartter syndrome, neuromuscular disorders and maternal hypercalcemia [13]. Although our study could not determine the etiology of polyhydramnios, we observed a trend toward more amniotic fluid with larger birth weights. It is unknown if this trend is caused by maternal diabetes or another etiologic factor that was not tested in the course of this study, and is not tested as part of routine antenatal care in the areas studied. Other factors, including sociodemographic and ethnic factors might contribute to polyhydramnios, but were not evaluated in this study. Additional investigation is warranted to determine the etiology of polyhydramnios in LMICs, and to determine the differential exposures that led to the higher incidence of polyhydramnios observed in this study in the DRC.

By demonstrating a high incidence of polyhydramnios in the DRC, we identify a critical gap in knowledge about the etiology of polyhydramnios and the site specific risk factors that contribute. Also, given the high rate of polyhydramnios, and the association with fetal malposition and obstructed labor, this represents an important publc health problem. In order to prevent pregnancy complications that result from antenatal conditions, like polyhydramnios, women need appropriate antenatal care to identify these conditions and appropriate referral for treatment to facilities with the capability to treat those conditions, including performing C-sections when warranted [14]. Despite the high incidence of polyhydramnios in the DRC compared to other sites, few C-sections were performed. In this study, the percentage of C-sections performed in the DRC was 1.5% compared to 13% in the other sites, which likely represents inadequate resources to perform C-sections in the area. In areas such as these, where resources for C-section are limited, direction of these resources to the highest risk patients might improve perinatal health. Therefore, the use of antenatal ultrasound to assist in the diagnosis of polyhydramnios may encourage appropriate referrals of high risk patients to delivery facilities capable of caring for these high risk patients.

## Conclusions

This study identified the incidence of polyhydramnios in LMICs, and confirmed its association with adverse pregnancy outcomes and neonatal mortality. We identified a high incidence of polyhydramnios in the DRC compared to other LMICs. These findings should be replicated, in order to better understand the higher than expected incidence of polyhydramnios in this area. If these findings are replicated, the identification of polyhydramnios during pregnancy could direct essential medical care to the women and infants most at risk for adverse pregnancy outcomes. In areas where resources for C-section are limited, a system that identifies women with polyhydramnios, and directs limited reources to these mothers, might reduce neonatal mortality rates in these high risk pregnancies. Identification of polyhydramnios during pregnancy, and appropriate treatment or referral could prevent subsequent adverse pregnancy outcomes and neonatal death. Our findings have underscored the importance of understanding the etiology of polyhydramnios in this environment and a case-control trial is underway to evaluate this finding.

## Data Availability

The datasets generated and analysed during the current study are not yet publicly available due to ongoing data analyses, but they will be available in the NICHD Data and Specimen Hub. Requests for data prior to the public release will be handled by the authors.

## Abbreviations

AFI: Amniotic fluid index
DRC: Democratic Republic of Congo
GA: Gestational age
GEE: Generalized estimating equations
LMIC: Low and low-middle income countries
MVP: Maximal vertical pocket
NICHD: National Institute of Health *Eunice Kennedy Shriver National* Institute of Child Health and Human Development
QA: Quality assurance
